# Chemical Speciation of Thorium in Marine Biogenic Particulate Matter

**DOI:** 10.1100/tsw.2004.4

**Published:** 2004-02-26

**Authors:** Katsumi Hirose

**Affiliations:** Geochemical Research Department, Meteorological Research Institute, 1-1 Nagamine, Tsukuba, Ibaraki 305-0052, Japan

**Keywords:** thorium, speciation, particulate matter, strong organic ligand, conditional stability constant

## Abstract

Concentrations of particulate thorium in seawater were determined together with the strong organic ligand (SOL) and uranium in particulate matter (PM). The concentrations of particulate Th in surface waters of the western North Pacific and the Sea of Japan ranged from 0.05 to 1.5 pM (1 x 10 M), and showed relatively large temporal and spatial variations. In order to chemically characterize the particulate Th in seawater, the relationship between particulate Th and SOL concentrations in surface PM was examined. The result reveals that particulate Th in surface PM was well correlated with the SOL concentration in PM. The concentrations of particulate Th in surface water were linearly related to those of particulate U. Mass balance analysis suggests that the dominant chemical form of Th(IV), as well as of U, in surface PM is a surface complex with the SOL in PM. Our findings suggest that the SOL in PM is a nonmetal-specific chelator originating from the cell surface of microorganisms.

## INTRODUCTION

Concentrations of metallic elements such as transition metals, lanthanides, and actinides in seawater are extremely low, generally ranging from nM to pM[1,2,3]. Some trace metals, e.g., Fe, Mn, Cu, Zn, Co, and Ni, are essential for growth of marine microorganisms, such as phytoplankton and bacteria. One major concern in the field of marine ecology is how planktonic organisms acquire and control the availability of trace metals[4]. In order to understand biological processes relating to metal acquisition in marine environments, we need to know the chemical forms of the metals, i.e., their chemical speciation, in seawater.

Marine chemists[5,6,7] have been studying the behavior of thorium isotopes in the ocean, although thorium is considered not to be a bioelement. One reason is that thorium isotopes ($^{230}$Th, $^{228}$Th, and $^{234}$Th) are used as tracers of biogeochemical processes, such as particle removal of trace metals from the water column[8,9,10]. In this case, it is implicitly assumed that a significant fraction of the thorium isotopes is associated with marine biogenic particulate matter. A typical example is that carbon export in the surface ocean, which is one of the most important biogeochemical processes in marine carbon cycling, is estimated by a method that is based on $^{238}U$-$^{234}Th$ disequilibria[11,12,13]. According to this method, the carbon export flux is calculated from product of the $^{234}Th$ flux and the POC/$^{234}Th$ ratio in the surface layer. However, the POC/$^{234}Th$ ratios in particulate matter (PM) vary according to the oceanographic and ecological conditions, i.e., particle size, depth, kind of microorganisms, and others[14]. Then the carbon export fluxes estimated from the $^{234}Th$ method have relatively large uncertainties. In order to elucidate carbon cycling related to biogeochemical processes in seawater, therefore, it is significant to understand the chemical interactions between particulate organic matter (POM) and thorium isotopes in seawater. However, there is little information on these interactions in oceanic waters because of the extremely low concentrations of Th[3].

Current chemical speciation studies of $^{234}Th$ in marine particulate and colloidal matter[15,16,17,18] suggest that $^{234}Th$ in particulate and colloidal matter is associated with binding sites stronger than EDTA, which are related to surface-active acid polysaccharides fraction in particulate and colloidal matter. However, chemical reactions between thorium and ligands are mostly responsible for an isotope with the largest amount of atoms; in fact, concentrations of radiogenic Th isotopes ($^{230}Th$, $^{228}Th$, and $^{234}Th$) in seawater are less than $10^{-16}$ M, especially, a maximum amount of $^{234}Th$ in seawater is only $0.2 \times 10^{-17}$ M. Therefore, it is important to have better understandings on chemical speciation of major thorium isotopes, i.e., $^{232}Th$. Recent speciation studies of trace elements[19,20,21,22] have revealed that strong organic ligands (SOL) are related to the interaction between trace metal ions (some transition metals such as Fe, Cu, and actinides [U and Pu]) and oceanic organic matter including both POM and dissolved organic matter (DOM). The SOL related to metal complexation in seawater is characterized chemically as a biogenic chelator with hard base properties, such as those of carboxylic acids, which are originally present on the cell-surface of marine microorganisms[23]. Therefore, it is likely that particle-surface complexation in PM is a viable candidate for the interactions between thorium (including $^{234}Th$) and POM.

In this paper, we describe the chemical speciation of thorium in PM based on mass balance analysis using the strong ligand concentration in PM.

## METHOD

### Sampling

Seawater samples were collected on board the RV Ryofu Maru, Keifu Maru, and Seifu Maru, owned by the Japan Meteorological Agency during the period from 1983–1997. Large volumes of surface seawater (100–1000 l) were collected on board using a pumping system. Water samples were filtered through a membrane filter (diameter of 293 mm) with a pore size of 0.45 μm (Millipore HA). Residues on the filter, including biogenic detritus, living and nonliving marine organisms, are defined as PM. Filter samples were stored frozen (–30°C) until analysis.

### Analytical Methods

Thorium analyses were carried out using alpha spectrometry. Eight samples were measured simultaneously with an alpha spectrometer comprised of eight solid-state detectors (CANBERRA Model PIPS) with a resolution of 25 kev FWHM at 5.486 MeV ($^{241}Am$) and a multichannel pulse height analyzer (CANBERRA).

All reagents were analytical grade unless otherwise noted. A stock solution of $Th^{4+}$ used for the SOL assay was prepared from reagent grade $ThO_2$ and 8 M hydrochloric acid. The $^{232}Th$ concentration in the stock solution was 10 Bq $l^{-1}$ (about 10 μM). An anion exchange resin, Dowex $1 \times 2$, 50–100 mesh

(BioRad Lab., USA), was used for ion exchange columns (column: 6 mm diameter and 150 mm long). The ion exchanger was successively washed with NaOH solution and diluted HCl solution before use.

In order to determine Th concentrations in PM, the residues on the filter were decomposed in concentrated $HNO_3$ and HCl on a hot plate. After digestion, the residue was dissolved in 8 M $HNO_3$ on a hot plate. The solution was passed through anion exchange resin column (nitrate form) and washed with 8 M $HNO_3$. Thorium adsorbed onto the anion exchange resin was eluted by 9 M HCl. The thorium fraction was dried up and dissolved in 8 M $HNO_3$ solution for further purification of the thorium fraction using anion exchange resin. The purified thorium was electrodeposited onto silver discs (diameter of 30 mm). The radioactivity counting was carried out by alpha spectrometry. The chemical yield (more than 95%) was confirmed by using known amounts of $^{230}$Th. The filter blank was less than 0.02 mBq for pieces of the membrane filter (293 mm). The detection limit of thorium ($^{232}$Th) in seawater (counting time: $10^6$ s) is ca 0.04 mBq $m^{-3}$. Urainum in PM was determined using the effluent of the Th separation column. Further details of the separation and analysis including electrodeposition of Th and U were described elsewhere[24].

The SOL concentrations in PM are deduced from the amount of thorium adsorbed on PM under the conditions of 0.1 M HCl based on results of complexation equilibrium studies between Th and a ligand in PM, in which Th forms only 1:1 complex with the ligand in PM under the acidic conditions[25]. The SOL measurements in PM were carried out at ambient temperature in 0.1 M HCl solution. A portion of dried filter samples was used for SOL determination. PM on the filter was equilibrated with a 20-ml Th solution in a 50-ml vessel for 24 h. After equilibration, PM was separated from the Th solution by filtration using a membrane filter with a pore size of 0.2 μm (47 mm diameter, Nucleopore). The residue was washed three times with 2 ml of 0.1 M HCl solution. The residue on the filter was decomposed with concentrated $HNO_3$ on a hot plate. The Th fraction was purified on anion exchange resin after dissolution in 8 M $HNO_3$. The Th fraction was eluted from the resin by 9 M HCl, and electrodeposited onto a silver disc. Th analysis was carried out by alpha spectrometry. The chemical yield for radiochemical analysis was determined with control runs by adding known Th concentrations. The SOL concentration in PM can be calculated using the following equation:

$$C_{SOL} = [ThL]\{1 + (K_{ThL,pH=1}[Th^{4+}])^{-1}\} \quad (1)$$

where [ThL] and $[Th^{4+}]$ are the concentrations of Th adsorbed onto PM and in solution, respectively, and $K_{ThL,pH=1}$ is the conditional stability constant for Th complexation with the strong ligand on PM. We used a value of $10^{6.6}$ $M^{-1}$ for $K_{ThL,pH=1}$ to calculate the SOL concentration in PM[25]. Under the experimental conditions, the uncertainty of the $K_{ThL,pH=1}$ value led to a systematic error of less than 10% for the SOL concentration. The SOL concentration in surface PM was reproducible within 5% for repeated runs.

## RESULTS

Concentrations of particulate thorium ($^{232}$Th) in oceanic waters are extremely low, i.e., equal or less than 1 pM[7,26,27]. We determined the concentrations of particulate Th in surface waters of the western North Pacific and the Sea of Japan during the period from 1983–1997. The results are summarized in Table 1, together with concentrations of particulate U and the SOL. The particulate thorium concentrations in surface waters of the western North Pacific and the Sea of Japan ranged from 0.05 ± 0.03 to 1.5 ± 0.2 pM (0.05 ± 0.03 to 1.4 ± 0.2 mBq $m^{-3}$). The particulate Th concentrations in surface waters of the western North Pacific were of the same order of magnitude as those in deep waters (0.1–1.1 pM)[7]. Relatively high concentrations of particulate Th were observed in May in the Sea of Japan and the equatorial Pacific. It is noteworthy that the concentrations of particulate Th in the western North Pacific show temporal and spatial variations.

**TABLE 1**
**Concentrations of Thorium, Uranium, and SOL in PM**

| Date | Location | | Part. U ($mBq\ m^{-3}$) | Part. Th ($mBq\ m^{-3}$) | SOL (nM) |
|---|---|---|---|---|---|
| **Western North Pacific** | | | | | |
| Jan. 1983 | 14° 00´ N | 136° 59´ E | 1.93 ± 0.29[a] | 0.56 ± 0.05[b] | |
| | 20° 00´ N | 136° 59´ E | 1.44 ± 0.22[a] | 0.25 ± 0.05[b] | |
| | 24° 00´ N | 137° 00´ E | 1.37 ± 0.21[a] | 0.49 ± 0.06[b] | |
| | 4° 31´ N | 137° 02´ E | 9.92 ± 0.9[a] | 1.60 ± 0.12[b] | |
| Jan. 1984 | 0° 02´ N | 136° 58´ E | 3.33 ± 0.37[a] | 0.60 ±0.06[b] | |
| | 5° 00´ N | 136° 56´ E | 4.74 ± 0.38[a] | 0.63 ± 0.08[b] | |
| | 9° 59´ N | 136° 57´ E | 5.63 ± 0.45[a] | 0.47 ± 0.07[b] | |
| | 14° 00´ N | 137° 00´ E | 10.7 ± 1.0[a] | 0.73 ± 0.08[b] | |
| July 1987 | 20° 00´ N | 155° 00´ E | 1.22 ± 0.10[b] | 0.32 ± 0.08[b] | |
| | 10° 00´ N | 155° 00´ E | 0.24 ± 0.05[b] | 0.05 ± 0.03[b] | |
| | 0° 00´ N | 155° 00´ E | 0.71 ± 0.08[b] | 0.16 ± 0.06[b] | |
| | 3° 00´ N | 137° 00´ E | 0.62 ± 0.07[b] | 0.18 ± 0.04[b] | |
| | 10° 00´ N | 137° 00´ E | 1.17 ± 0.10[b] | 0.13 ± 0.03[b] | |
| | 14° 00´ N | 137° 00´ E | 0.74 ± 0.09[b] | 0.16 ± 0.06[b] | |
| | 21° 00´ N | 137° 00´ E | 0.73 ± 0.08[b] | 0.18 ± 0.04[b] | |
| | 28° 00´ N | 137° 00´ E | 0.88 ± 0.08[b] | 0.15 ± 0.04[b] | |
| | 34° 00´ N | 137° 00´ E | 0.76 ± 0.08[b] | 0.17 ± 0.04[b] | |
| June 1991 | 32° 00´ N | 140° 15´ E | 2.90 ± 0.42[c] | | 3.41 ± 0.19[c] |
| | 30° 00´ N | 140° 07´ E | 2.13 ± 0.38[c] | | 2.30 ± 0.13[c] |
| | 28° 00´ N | 137° 00´ E | 4.96 ± 0.35[c] | 0.42 ± 0.09[b] | 5.84 ± 0.32[c] |
| | 14° 00´ N | 137° 00´ E | 2.79 ± 0.22[c] | 0.32 ± 0.08[b] | 3.37 ± 0.16[c] |
| | 10° 00´ N | 137° 00´ E | 6.07 ± 0.36[c] | 1.02 ± 0.13[b] | 7.31 ± 0.42[c] |

| | | | | | |
|---|---|---|---|---|---|
| | 3° 00´ N | 144° 00´ E | 8.24 ± 0.58[c] | 1.09 ± 0.13[b] | 9.47 ± 0.42[c] |
| | 7° 00´ N | 144° 00´ E | 2.17 ± 0.15[c] | 0.38 ± 0.09[b] | 3.22 ± 0.16[c] |
| | 22° 00´ N | 144° 00´ E | 3.29 ± 0.23[c] | 0.41 ± 0.05[b] | 4.63 ± 0.21[c] |
| Jan. 1993 | 14° 00´ N | 137° 00´ E | | 0.12 ± 0.03[b] | 1.08 ± 0.06[b] |
| | 5° 00´ N | 137° 00´ E | | 0.22 ± 0.05[b] | 3.03 ± 0.15[b] |
| Jan. 1997 | 34° 00´ N | 137° 00´ E | | 0.44 ± 0.12[b] | 5.04 ± 0.37[b] |
| | 30° 00´ N | 137° 00´ E | | 0.52 ± 0.20[b] | 6.77 ± 0.46[b] |
| | 20° 00´ N | 137° 00´ E | | | 3.22 ± 0.28[b] |
| | 15° 00´ N | 137° 00´ E | | 0.27 ± 0.09[b] | 2.72 ± 0.23[b] |
| | 5° 00´ N | 137° 00´ E | | 0.56 ± 0.14[b] | 4.58 ± 0.31[b] |
| **Sea of Japan** | | | | | |
| May 1993 | 38° 09´ N | 134° 27´ E | 11.3 ± 0.6[c] | 1.39 ± 0.15[b] | 13.5 ± 0.5[c] |
| | 39° 25´ N | 133° 25´ E | 10.5 ± 0.7[c] | 1.25 ± 0.15[b] | 11.4 ± 0.6[c] |

[a] Hirose and Sugimura, 1991.
[b] This work.
[c] Hirose, 1995.

The SOL concentrations in surface PM ranged from 1.08 ± 0.06 to 13.5 ± 0.5 nM, which are of the same order of magnitude as in previous results[20,21]. The particulate U concentrations in surface waters of the western North Pacific and marginal seas ranged from 0.08 ± 0.02 to 3.8 ± 0.2 pM (0.24 ± 0.05 to 11.3 ± 0.6 mBq $m^{-3}$), which were four orders of magnitude lower than those of dissolved U (15 nM)[20,24,27]. In contrast to dissolved U, particulate U in the western North Pacific surface water showed large temporal and spatial variations, similar to those of particulate Th[24].

## DISCUSSION

### Interaction Between Thorium and SOL in PM

In order to elucidate factors controlling variations of particulate Th in surface waters, it is important to have a better understanding of thorium-particle interactions in seawater, that is, the chemical speciation of particulate thorium. Sequential leaching studies[26] suggest that $^{232}$Th in PM forms complexes with an organic ligand. To understand the chemical characteristics of particulate Th, the relationship between thorium and the SOL in PM is examined based on thermodynamic considerations. The application of mass balance analysis to particulate U and Pu[20,22] is possible in seawater because the chemical compositions of seawater, i.e., the concentrations of major ions and pH, are almost constant. Thermodynamic parameters, such as conditional stability constants, partition coefficients, and side reaction coefficients, are useful tools to characterize the chemical forms of thorium. Previous studies[19,23,25] revealed that the SOL, which is found in PM, DOM, and marine microorganisms, is related to the complexation with hard metals such as Th, U, Fe(III), and Pu(IV). It, therefore, is important to examine the relationship between Th species and the SOL based on a mass balance analysis.

Recent knowledge of thorium in seawater[3,7,29,30,31] is that concentration levels of dissolved Th in oceanic water (0.02–2 pM) are supersaturated with regard to the dissolution of its inorganic salts (the solubility of thorianite in seawater is about 0.01 pM)[32]. It must be noted that there are significant uncertainties on solubility of inorganic Th compounds in seawater[33,34,35]. The excess Th is presumed to be present as organic complexes and/or colloidal forms[36,37,38]. Therefore, it may be reasonable to assume that the free ion concentration of Th, $[Th^{4+}]$, in seawater is controlled by the dissolution equilibrium of thorium hydroxide. In this case, according to the mass balance analysis[19], the following equilibrium is established between Th and SOL in PM.

$$Th^{4+} + L_p = ThL_p \text{ and } Th^{4+} + 4OH^- = Th(OH)_{4,s} \quad (2)$$

The conditional stability constant of the Th(IV) complex is written as follows:

$$K_{ThL,SW} = [ThL_p][Th^{4+}]^{-1}[L_p]^{-1} \quad (3)$$

where $[L_p]$ is the concentration of the SOL unbound with metals, and $[Th^{4+}]$ is the concentration of free Th(IV) ion, which is controlled by the solubility product of Th hydroxide ($=[Th^{4+}][OH^-]^4$), due to its extremely low value of $10^{-46.6}$[32]. The concentration of free Th(IV) ion ($=10^{-25.8}$) is maintained at a constant level in the ocean because of constant pH and ionic composition of seawater (pH = 8.2, I = 0.7). In this case, the concentrations of particulate Th in surface waters of the western North Pacific, $C_{Th,p}$, are linearly related to the SOL concentration in PM, $C_{SOL}$, as shown by the following equation:

$$C_{Th,p} = [ThL_p] = K_{ThL,SW}\,[Th^{4+}]a_{L(Mi)}^{-1}C_{SOL} \quad (4)$$

where $C_{SOL}$ is equal to $[L_p]\,a_{L(Mi)}$; $a_{L(Mi)}$ is the side reaction coefficient of the SOL. A plot of $C_{Th,p}$ to $C_{SOL}$ is shown in Fig. 1, which shows a good linear relationship between $C_{Th,p}$ and $C_{SOL}$. The result suggests that Th is associated with the SOL in PM and that the contents of particulate Th are controlled by the SOL concentrations in PM. The slope of the linear regression ($=1.1 \times 10^{-4}$) is equal to the value of $K_{ThL,SW}$ $[Th^{4+}]a_{L(Mi)}^{-1}$. The conditional stability constant of the Th(IV) complex in PM is estimated from the free Th(IV) ion concentration and the side reaction coefficient of the SOL in PM. Our previous studies of natural chelators in seawater[19,21,22,23] introduced a hypothesis that the SOL found in PM, DOM, and organisms is classified as a DTPA (diethylenetrinitrilopenta-acetic acid) type. According to the coordination model[19], the concentration of the DTPA type organic ligand not bounded with metals was estimated to be around 1% of the total, which means the side reaction coefficient of the ligand is about $10^2$. Therefore, the conditional stability constant of Th(IV) complex in PM under the conditions of seawater is calculated to be about $10^{23.8}$ $M^{-1}$. Interestingly, the conditional stability constant of Th complex in PM is the same order of magnitude as that of Pu(IV)[22]. The finding reveals that most of the thorium in PM is present as the complex with the SOL in PM and that the concentration of particulate Th in surface waters is primarily controlled by that of the SOL in PM.

There is a large difference between the conditional stability constants of the Th complex in seawater and that in 0.1 M HCl. Its cause is due to side-reactions of protonation to the SOL in PM, because the SOL possesses several weak acid residues. The conditional stability constant of the Th complex is given as follows, using the side reaction coefficients of the SOL.

$$K_{ThL,SW} = K_{ThL}\,a_{L(H,SW)} \text{ and } K_{ThL,pH=1} = K_{ThL}\,a_{L(H,pH=1)} \quad (5)$$

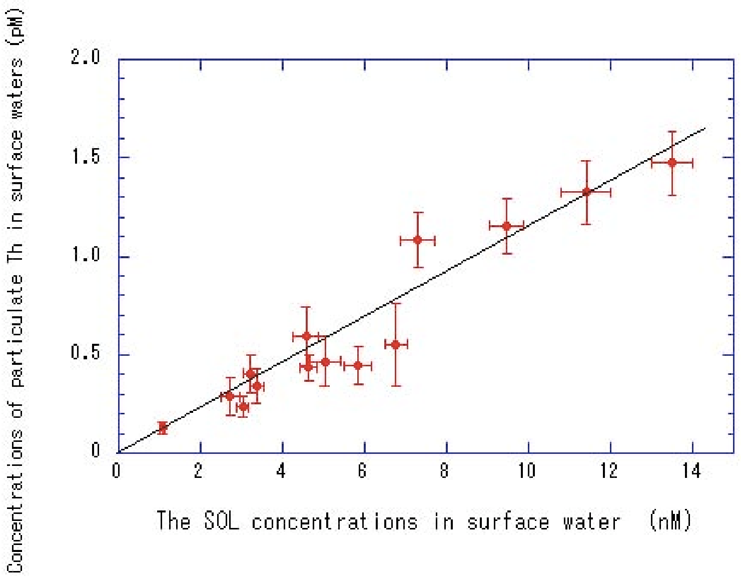

**FIGURE 1.** Relationship between the concentrations of particulate Th and the SOL in surface waters of the western North Pacific and the Sea of Japan.

where $K_{ThL}$ is the stability constant of the Th complex with the SOL. We calculated a ratio of the value of $K_{ThL,pH=1}$ to $K_{ThL,SW}$ for the SOL. To compare with the ratio of known chelators (aminopolycarboxylic acids), the ratios (= $K_{ThL,SW}/K_{ThL,pH=1}$) are calculated from protonation constants of known chelators[39]. This calculation resulted in the following order: L-alanine (8.5) < citric acid (9.3) < NTA (9.6) < EDTA (15.1) < CyDTA (15.9) < the SOL (17.3) < DTPA (19.8) < TTHA (25.5), where the value in parenthesis is the logarithm of the ratio ($K_{ThL,SW}/K_{ThL,pH=1}$). This ratio primarily depends on the number of acid sites in chelators. As a result, it is likely that the SOL in PM is a polydentate ligand, which may be a chelator with five acid sites.

## The Relationship Between Particulate Th and U

Uranium in PM forms complexes with the strong organic ligands in PM[20,24,26], although particulate uranium in seawater is a minor species compared to dissolved uranium. One can expect the following relationship between the concentrations of particulate Th ($C_{Th,p}$) and particulate U ($C_{U,p}$) if the strong ligand in PM reacting with thorium is also able to react with uranium.

$$C_{Th,p} = (K_{ThLp,SW}/K_{ULp,SW})([UO_2^{2+}]^{-1} [Th^{4+}])C_{U,p} \quad (6)$$

where $K_{Ulp,SW}$ (=$[UO_2L_p][UO_2^{2+}]^{-1}[L_p]^{-1}$) is the conditional stability constant of the uranium complex with the SOL in PM, and $[UO_2^{2+}]$ is the concentration of free uranyl ion. If the free ion concentration of uranium and thorium is kept constant in surface waters, we obtain a linear relationship between particulate thorium and uranium. In seawater, uranium, which is dissolved as a stable carbonato complex ($UO_2[CO_3]_3^{4-}$)[40], is conservative[28]. Therefore, the concentration of free uranyl ion (=$10^{-16.1}$) is constant in marine environments. On the other hand, the free thorium ion concentration in seawater is maintained at a constant level due to solubility equilibrium as discussed in the previous section. In this case, we expect a linear relationship between the concentrations of particulate uranium and particulate thorium. Fig. 2 shows the logarithmic relationship between concentrations of uranium and thorium in the oceanic PM. The logarithmic concentration of particulate uranium is linearly related to that of particulate thorium with a slope of unity. An intercept (=0.5) is equal to the value of $(K_{ThL,SW}/K_{ULp,SW})([UO_2^{2+}]^{-1}[Th^{4+}])$. The conditional stability constant of the uranium complex can be estimated to be $10^{14.1}$ $M^{-1}$ from the values of the free uranium and thorium concentrations and the conditional stability constant of thorium with the SOL. This value is in good agreement with the previous estimate[24]. The conditional stability constants of metals with the SOL under the conditions of seawater show the following sequence: Pu(IV) ≈ Th > Fe(III) >> U(VI) > Cu(II) > Zn. These results suggest that the SOL in PM, which has higher affinity to four valent actinides, such as Th(IV) and Pu(IV), is a kind of hard base and nonspecific for trace metals. It may be deduced that a dominant factor controlling concentrations of several trace metals, including radionuclides, in oceanic PM is the concentration of the SOL in PM. Therefore, a major part of the spatial and temporal variations of particulate Th results in the variations of the SOL in PM, which may reflect biological activity in the marine environment.

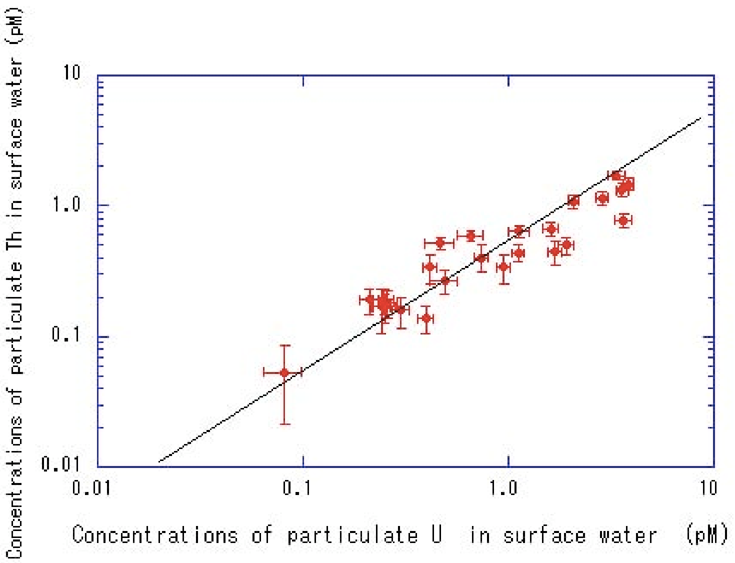

**FIGURE 2**. Relationship between the concentrations of particulate Th and particulate U in surface waters of the western North Pacific and the Sea of Japan.

## CONCLUSIONS

Mass balance analysis suggests that most of the thorium in PM is associated with the SOL produced by marine microorganisms. The SOL in PM can react with transition metals and actinides under the conditions of seawater, although the conditional stability constants of metal complexes depend on valent state of metal ion, some side reactions with inorganic ligands including hydrolysis. Therefore, the concentrations of particulate Th and U in surface waters are primarily controlled by the SOL concentration in PM, which may be related to productivity of marine microorganisms and biological properties such as cell size[25].

This biogenic organic ligand in PM, which has a higher affinity to hard acids (e.g., Pu[IV], Th[IV], Fe[III], and others), is nonspecific for metal ions, and behaves like a aminopolycarboxylic acid. This ligand may be related to the accumulation of trace metals onto the cell-surface of oceanic microorganisms.

## ACKNOWLEDGMENTS

The author thanks the captain and the crew members of RV Ryofu Maru, Keifu Maru, and Seifu Maru and the staff members of Geochemical Research Department for on-board sampling. The author would like to thank anonymous reviewers for the constructive comments and suggestion.
